# Estrogens regulate neuroinflammatory genes via estrogen receptors α and β in the frontal cortex of middle-aged female rats

**DOI:** 10.1186/1742-2094-8-82

**Published:** 2011-07-20

**Authors:** Miklós Sárvári, Erik Hrabovszky, Imre Kalló, Norbert Solymosi, Kinga Tóth, István Likó, János Széles, Sándor Mahó, Béla Molnár, Zsolt Liposits

**Affiliations:** 1 Laboratory of Endocrine Neurobiology, Institute of Experimental Medicine, Hungarian Academy of Sciences, Szigony utca 43, Budapest, 1083 Hungary; 2 Faculty of Information Technology, Pázmány Péter Catholic University, Práter utca 50/A, Budapest, 1083 Hungary; 3 Department of Physics of Complex Systems, Eötvös Loránd University, Pázmány Péter sétány 1/A, Budapest, 1117 Hungary; 4 Second Department of Medicine, Semmelweis University, Szentkirályi utca 46, Budapest, 1088 Hungary; 5 Pharmacology and Drug Safety Research, Gedeon Richter Plc., Gyömrői út 19-21, Budapest, 1103 Hungary; 6 API Research and Development, Gedeon Richter Plc., Gyömrői út 19-21, Budapest, 1103 Hungary; 7 Molecular Medicine Research Unit, Hungarian Academy of Sciences, Szentkirályi utca 46, Budapest, 1088 Hungary

## Abstract

**Background:**

Estrogens exert anti-inflammatory and neuroprotective effects in the brain mainly via estrogen receptors α (ERα) and β (ERβ). These receptors are members of the nuclear receptor superfamily of ligand-dependent transcription factors. This study was aimed at the elucidation of the effects of ERα and ERβ agonists on the expression of neuroinflammatory genes in the frontal cortex of aging female rats.

**Methods:**

To identify estrogen-responsive immunity/inflammation genes, we treated middle-aged, ovariectomized rats with 17β-estradiol (E2), ERα agonist 16α-lactone-estradiol (16α-LE2) and ERβ agonist diarylpropionitrile (DPN), or vehicle by Alzet minipump delivery for 29 days. Then we compared the transcriptomes of the frontal cortex of estrogen-deprived *versus *ER agonist-treated animals using Affymetrix Rat230 2.0 expression arrays and TaqMan-based quantitative real-time PCR. Microarray and PCR data were evaluated by using Bioconductor packages and the RealTime StatMiner software, respectively.

**Results:**

Microarray analysis revealed the transcriptional regulation of 21 immunity/inflammation genes by 16α-LE2. The subsequent comparative real-time PCR study analyzed the isotype specific effects of ER agonists on neuroinflammatory genes of primarily glial origin. E2 regulated the expression of sixteen genes, including down-regulation of complement C3 and C4b, Ccl2, Tgfb1, macrophage expressed gene Mpeg1, RT1-Aw2, Cx3cr1, Fcgr2b, Cd11b, Tlr4 and Tlr9, and up-regulation of defensin Np4 and RatNP-3b, IgG-2a, Il6 and ER gene Esr1. Similar to E2, both 16α-LE2 and DPN evoked up-regulation of defensins, IgG-2a and Il6, and down-regulation of C3 and its receptor Cd11b, Ccl2, RT1-Aw2 and Fcgr2b.

**Conclusions:**

These findings provide evidence that E2, 16α-LE2 and DPN modulate the expression of neuroinflammatory genes in the frontal cortex of middle-aged female rats via both ERα and ERβ. We propose that ERβ is a promising target to suppress regulatory functions of glial cells in the E2-deprived female brain and in various neuroinflammatory diseases.

## Background

The complex interactions between the immune and central nervous systems govern the innate immune responses in the brain [1]. Microglial cells survey their environment through continuous remodeling of cellular processes [2]. These cells respond to injury or infection and induce a variety of secondary responses including activation of astrocytes [3] and migration of peripheral immune cells into the brain [4,5]. The activation of glial cells and recruitment of immune cells subserve the brain homeostasis. Estrogens modulate the function of many cell types of the immune [6] and the central nervous systems [7,8]. In females, E2 levels drop abruptly at the time of menopause resulting in a low grade of systemic inflammation which can be prevented by chronic treatment with low dose of E2 [9]. E2 modulates inflammatory processes in models of human diseases such as arthritis [10], systemic lupus erythematosus, Alzheimer disease [11] and multiple sclerosis [12]. In the rat brain, E2 suppresses activation of microglia, recruitment of blood-derived monocytes, and expression of C3 receptor and matrix metalloproteinase-9 after intracerebroventricular injection of LPS [13]. E2 also inhibits the expression of pro-inflammatory cytokines IL1β and TNFα in LPS-treated primary astrocytes [14]. These studies indicate that E2 may regulate both microglia and astrocyte functions related to inflammation.

The effects of E2 are primarily mediated by ERα and ERβ which are members of the nuclear receptor superfamily of ligand-activated transcription factors [15]. ERα and ERβ regulate gene expression through multiple mechanisms. Via a classic mode of action, ERs can induce transcription upon binding to estrogen-responsive elements in target gene promoters. They can also modulate transcription via interfering with other promoter-bound transcription factors, or via influencing a variety of intracellular signaling pathways [16]. In the frontal cortex, E2 may alter gene transcription directly via ERs in inhibitory interneurons [17], astrocytes [18] and microglia [13,19]. However, the knowledge on estrogenic regulation of neuroinflammatory genes is limited in the cerebral cortex of middle-aged females. In a rodent menopausal model, we have recently described changes of the cortical transcriptome as a result of E2 replacement [20]. We have identified some immunity genes encoding complement (C) proteins and MHC antigens among the genes with the highest fold change. Down-regulation of these genes is in line with the anti-inflammatory activity of E2 in neuroinflammatory disease models [11-13].

To identify estrogen-responsive neuroinflammatory genes in the frontal cortex of middle-aged female rats, we compared the transcriptomes of ovariectomized and ERα agonist-treated animals using oligonucleotide microarrays. Based on the results of our microarray analysis and on the knowledge regarding the expression profile of glial cells, we selected a set of potential estrogen target genes of primarily glial origin. Then we examined the transcription of these genes involved in the recognition of danger- and pathogen-associated signals, cellular defense, phagocytosis, neuron-microglia communication and immune regulation after chronic treatments with E2, ERα agonist 16α-LE2 [21] and ERβ agonist DPN [22]. We demonstrated that these ER agonists regulate the transcription of a large number of neuroinflammatory genes in the frontal cortex of middle-aged female rats.

## Methods

### Chemicals

3,17β-dihydroxy-19-nor-17α-pregna-1,3,5(10)-triene-21,16α-lactone (16α-LE2) was originally designed, synthesized and patented by Schering AG [23]. This compound was re-synthesized in the Laboratory of Steroid Chemistry at Gedeon Richter Plc. NMR spectra (Varian NMR System 300) and melting points were identical to published data [23]. E2 and DPN were purchased from Sigma (St. Louis, MO) and Tocris (Ellisville, MO), respectively.

### Experimental animals and treatments

**Figure 1:**
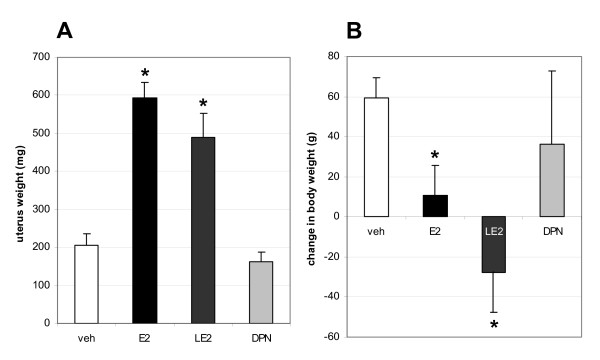
**The effects of estrogen replacements on uterus weight (A) and body weight (B)**. E2 and ERα agonist 16α-lactone-estradiol (LE2) changed the uterus weight significantly (p values for E2 and LE2 were p = 0.0001 in both cases), while DPN had no effect. In a similar way, E2 and LE2 decreased body weight significantly (p values for E2 and LE2 were p = 0.0035 and p = 0.0001, respectively). Change in body weight represents the weight difference between the weight of the animal before and after the chronic treatment. Asterisks mark statistically significant changes compared to vehicle. Error bars correspond to standard deviations.

Female, middle-aged retired breeder Harlan-Wistar rats were purchased from Toxicoop (Budapest, Hungary). Animals were housed individually in the animal care facility of Institute of Experimental Medicine (IEM) on a 12 h light/12 h dark cycle, and with unrestricted access to phytoestrogen-free rodent diet (Harlan Teklad Global Diets, Madison, WI) and tap water. At the age of 13 months, the rats were deeply anesthetized and ovariectomized bilaterally. Ten days later, Alzet 2004 minipumps (DURECT, Cupertino, CA) filled with 16α-LE2 (3,33 mg/ml in propylene-glycol, n = 6) and vehicle (n = 6, control group) were implanted subcutaneously for 29 days. Concentration of 16α-LE2 was calculated to produce a release rate of 20 μg/d [21]. For further replacement experiments, Alzet 2004 minipumps were filled either with E2 (0,333 mg/ml in propylene-glycol, n = 6) or DPN (3,33 mg/ml in propylene-glycol, n = 6) and were implanted for 29 days. Concentrations were calculated to produce a release rate of 2 μg/d and 20 μg/d, respectively [21]. Body weight and uterus weight were measured to follow the peripheral effects of the treatments (Figure 1). For the preparation of the frontal cortex the same protocol was followed as published earlier [24]. Protocols were approved by the Animal Welfare Committee of IEM (No.: A5769-01). Experiments were carried out in accordance with the legal requirements of the European Community (Decree 86/609/EEC).

### Total RNA isolation from the cerebral cortex

Total RNA was isolated from the frontal cortex using the RNeasy Lipid Tissue Mini Kit (QIAGEN, Hilden, Germany). RNA analytics included A260 nm/A280 nm readings using a Nanodrop Spectrophotometer and capillary electrophoresis using Agilent 2100 Bioanalyzer (Santa Clara, CA). All RNA samples displayed RNA integrity numbers (RIN) above 8.2.

### Expression profiling using Rat 230 2.0 Expression Arrays

One-cycle target labeling, hybridization, staining and scanning were carried out as described earlier [24]. In brief, preparation of poly-A RNA controls (spike-in controls), first and second strand cDNA synthesis, cleanup, *in vitro *transcription labeling, cleanup of biotin-labeled cRNA and fragmentation were carried out according to the Affymetrix technical manual. Fragmented cRNA was hybridized for 16 h to Affymetrix Rat 230 2.0 Expression Array (Santa Clara, CA). Arrays were washed, and stained with phycoerythrin-conjugated streptavidin (Molecular Probes, Eugene, OR). Fluorescence intensities were determined using the GCS 3000 confocal laser scanner (Affymetrix). Scanned images were analyzed using programs resident in GeneChip^® ^Operating System v1.2 (GCOS Affymetrix).

### Data analysis

For data analysis, we followed the same protocol as before [20]. In brief, for quality control we used the affyQCReport package. Raw microarray data were pre-processed for analysis by GCRMA [25]. After outlier identification [26], linear models combined with empirical Bayesian methods were applied [27] and the raw fold change values were used to select differentially expressed genes [28]. P-values were adjusted by the FDR-based method [29]. In gene set enrichment analysis, KEGG (Kyoto Encyclopedia of Genes and Genomes) pathways [30] were used as collaborator gene sets, analyzed by a recently developed method [31]. In all statistical and data mining work Bioconductor packages [32] in R-environment [33] were used.

### Quantitative real-time PCR

Custom TaqMan low density arrays (TLDA) were designed to confirm microarray results and to study in depth the regulation of microglia-related genes by quantitative real-time PCR. Microfluidic cards (Applied Biosystems, Santa Clara, CA) were preloaded by the manufacturer with selected inventoried assays for the genes of our interest and for five potential house-keeping genes including 18S rRNA, Gapdh, glucuronidase beta (Gusb), hypoxanthine guanine phospho-ribosyl-transferase (Hprt1) and peptidyl-prolyl isomerase A (Ppia). Each assay consisted of a FAM dye-labeled TaqMan MGB probe and two PCR primers. Every assay had been optimized by the manufacturer to run under universal thermal cycling conditions with a final reaction concentration of 250 nM for the probe and 900 nM for each primer. Reverse transcription and real-time PCR were run as described earlier [20]. RealTime StatMiner (Integromics, Granada, Spain) software and relative quantification against calibrator samples (ΔΔCt) were used for analysis of Applied Biosystems TaqMan gene expression assays. Five house-keeping genes were applied on the TLDA card as potential internal controls. To find the most stable endogenous controls, the nonfinder stability scoring method [34] was used. A computed internal control corresponding to the geometric mean of Ct values of Gapdh, Hprt1 and Ppia was used for subsequent ΔCt calculation [35]. Relative quantity (RQ) represents the expression of a given gene in response to a treatment compared to basal (control) expression.

## Results

### Expression profiling revealed numerous ERα agonist-regulated immunity genes in the middle-aged female neocortex

**Figure 2:**
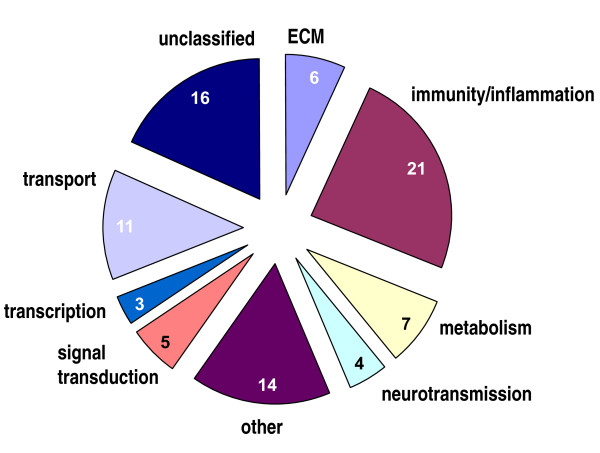
**Microarray analysis revealed 87 ERα agonist-regulated genes which were categorized based on function**. ERα agonist-regulated genes included twenty-one immunity/inflammation genes. The cluster contained S100 calcium-binding (S100a9, S100a8) and defense proteins (RatNP-3b, Np4, Defa, Camp), complement C3 and Serping1, Ig chains (Igha, IgG-2a, Igj), mast cell proteases (Mcpt8, Mcpt9) and MHC antigens (RT1-Aw2, RT1-N1).

Oligonucleotide microarrays were used to study the effects of the selective ERα agonist 16α-LE2 on the cortical gene expression profile of middle-aged, ovariectomized rats. Differences between the cortical transcriptomes of vehicle- and ERα agonist-treated animals were evaluated, and the top100 ERα agonist-regulated probe sets, i.e. probe sets with the highest absolute fold change, were identified. The 100 probe sets encoded 87 ERα agonist-responsive genes, which were categorized based on function (Figure 2). A characteristic feature of the gene list was the high proportion (24%) of genes related to immunity/inflammation. Transcriptional regulation of the 21 immunity genes included down-regulation of complement C3 and Serping1, MHC genes (Cd74, RT1-Aw2, RT1-N1), Fcgr2b, and up-regulation of antimicrobial peptide (RatNP-3b, Np4, Defa, Camp) and S100 protein genes (S100a9, S100a8), Ig chains (IgG2a, Igh-1a, Igj, Igha), mast cell proteases (Mcpt9, Mcpt8), Fcnb, Prg2 and Lrrc8a (Table 1).

**Table 1:** Confirmation of the ERα agonist-regulated immunity/inflammation genes by quantitative real-time PCR.

GENE		MICROARRAY		REAL-TIME PCR	
Symbol	Name	probe set	FC	TaqMan ID	RQ
***S100a9***	S100 calcium binding protein A9	1387125_at	3.655	Rn00585879_m1	2.029
***S100a8***	S100 calcium binding protein A8	1368494_at	3.458	Rn00587579_g1	2.558
***RT1-Aw2***	RT1 class Ib	1388202_at	0.291	Rn03034964_u1	0.658
***RatNP-3b***	defensin ratNP-3 precursor	13700791_at	3.317	Rn01478511_gH	10.568
***IgG-2a***	immonoglobulin G	1370394_at	3.204	Rn01429839_g1	9.257
***Np4***	defensin NP-4 precursor	1370470_at	3.031	Rn00597762_g1	2.268
***Defa***	defensin, alpha 5, Paneth cell-specific	1387943_at	2.732	Rn02607254_g1	32.763
***Prg2***	proteoglycan 2	1387633_at	2.412	Rn00581137_m1	1.257
***Camp***	cathelicidin antimicrobial peptide	1393603_at	2.297	Rn01446021_g1	1.521
***Mcpt9***	mast cell protease 9	1368501_s_at	2.235	Rn00755366_g1	5.825
***Fcgr2b***	Fc fragment of IgG, receptor	1371079_at	0.451	Rn00598391_m1	0.649
***Igh-1a***	immunoglobulin heavy chain	1388272_at	2.144		
***Cd74***	CD74	1367679_at	0.473	Rn00565062_m1	0.685
***Fcnb***	ficolin beta	1387378_at	2.071	Rn00586231_m1	1.922
***Mcpt8***	mast cell protease 8	1369586_at	2.056	Rn01789238_g1	3.784
***Igj***	immunoglobulin joining chain	1383163_at	1.945	Rn01768305_m1	1.204
***Igha***	immunoglobulin heavy chain, alpha	1371262_at	1.866		
***RT1-Aw2***	RT1 class Ib	1388203_x_at	0.536	Rn03034964_u1	0.658
***Serping1***	C1-Inhibitor	1372254_at	0.559	Rn01485600_m1	0.690
***C3***	complement C3	1368000_at	0.559	Rn00566466_m1	0.743
***RT1-N1***	RT1 class Ib, locus N1	1387839_at	0.570	Rn00561858_m1	0.654
***Lrrc8a***	leucine rich repeat containing 8 family	1382920_at	1.670		
					

Transcriptional regulation of twenty-one immunity genes determined by the top100 probe sets of microarray analysis was confirmed by real-time PCR. FC, fold change; RQ, relative quantity.

Eighteen immunity genes were selected to confirm the changes in their expression after ERα agonist treatment by quantitative real-time PCR. All transcriptional changes were confirmed (Table 1). The results provided proof for the profound transcriptional regulation of immunity/inflammation genes by 16α-LE2.

Pathway analysis supported the potent immunomodulatory effects of 16α-LE2 in the aging cortex. The list of the top 20 pathways contained eight immunity-related KEGG pathways including graft-versus-host disease, autoimmune thyroid disease, allograft rejection, hematopoietic cell lineage, C and coagulation cascades, cytokine-cytokine receptor interaction, systemic lupus erythematosus and Jak-STAT signaling pathway (Table 2).

**Table 2:** Pathway analysis using Tian's method identified ERα agonist-regulated pathways related to immunity/inflammation.

rank	pathway	set size	percent up	average (NTk, NEk)
**1**	**graft-versus-host disease**	**67**	**9**	**7.2**
**2**	**autoimmune thyroid disease**	**73**	**8**	**7.5**
**3**	**allograft rejection**	**68**	**9**	**7.8**
4	cell adhesion molecules	189	18	8.0
5	retinol metabolism	64	11	9.5
6	type I diabetes mellitus	77	16	11.2
**7**	**hematopoietic cell lineage**	**101**	**18**	**13.5**
8	neuroactive ligand-receptor interaction	335	16	13.8
9	pantothenate and CoA biosynthesis	11	27	14.5
**10**	**C and coagulation cascades**	**92**	**13**	**14.5**
11	androgen and estrogen metabolism	33	15	16.5
12	Parkinson's disease	139	69	16.5
**13**	**cytokine-cytokine receptor interaction**	**210**	**23**	**20.5**
**14**	**systemic lupus erythematosus**	**92**	**22**	**22.2**
15	caffeine metabolism	10	10	25.5
16	metabolism of xenobiotics by cytochrome P450	48	15	26.2
17	ECM-receptor interaction	101	24	26.2
**18**	**Jak-STAT signaling pathway**	**172**	**28**	**27.2**
19	drug metabolism - other enzymes	38	24	27.5
20	basal cell carcinoma	68	21	29.0

The analysis identified eight immune-related KEGG pathways (in bold) regulated by selective ERα agonist 16α-LE2. In the analysis, KEGG pathways were used as collaborator gene sets. Using the Tian's method [31], the relationship between gene sets and treatment is quantified by two statistics (*T_k _*and *E_k_*) formulating the two hypotheses: i) the gene in a gene set shows the same pattern of associations with the treatment compared with the rest of the genes, ii) the gene set does not contain any genes whose expression levels are associated with the treatment, respectively. KEGG pathways were ranked by the mean of normalized statistics (*NT_k _*and *NE_k_*). Set size is the number of genes, percent up is the number of up-regulated genes in the KEGG pathway. C, complement.

### Examination of the estrogenic regulation of neuroinflammatory genes

Next, we selected genes involved in the recognition of danger- and pathogen-associated signals, phagocytosis, neuron-microglia communication and immunoregulation. Although some genes were expressed in both neurons and glia, most of the selected genes were predominantly expressed in glial cells, several of them were specific for microglia. We examined the effects of E2, 16α-LE2 and ERβ agonist DPN on the transcription of these genes.

### Genes regulated by E2

We identified sixteen E2-dependent changes by quantitative real-time PCR (Table 3). The E2-regulated genes included defensin Np4 and RatNP-3b, S100 calcium binding protein gene S100a8, C3 and C4b, Ig chain IgG-2a, lymphokines Ccl2, Il6 and Tgfb1, MHC gene RT1-Aw2, macrophage expressed gene Mpeg1, ERα gene Esr1, phagocytic receptors Fcgr2b and Itgam/Cd11b, and toll-like receptors Tlr4 and Tlr9.

**Table 3:** Real-time PCR revealed that E2 regulated the transcription of neuroinflammatory genes in the frontal cortex of middle-aged female rats.

Symbol	Gene name	TaqMan ID	RQ(E2)	p
**complement**				
*C3*	complement C3	Rn00566466_m1	0.70	0.02
*C4b*	complement C4	Rn00709527_m1	0.67	0.07
**defensin**				
*Np4*	defensin NP-4 precursor	Rn00597762_g1	2.56	0.08
*RatNP-3b*	defensin ratNP-3 precursor	Rn01478511_gH	13.27	0.06
**Ig chain**				
*IgG-2a*	immonoglobulin G	Rn01429839_g1	8.23	0.10
**lymphokine**				
*Ccl2*	chemokine (C-C) ligand 2	Rn00580555_m1	0.52	0.06
*Il6*	interleukin-6	Rn00561420_m1	2.82	0.04
*Tgfb1*	transforming growth factor beta 1	Rn00572010_m1	0.78	0.01
**MHC**				
*RT1-Aw2*	RT1 class Ib	Rn03034964_u1	0.61	0.10
**miscellaneous**				
*Cx3cr1*	fractalkine receptor	Rn00591798_m1	0.82	0.04
*Mpeg1*	macrophage expressed gene 1	Rn02769865_s1	0.61	0.01
**nuclear receptor**				
*Esr1*	estrogen receptor-α	Rn00562166_m1	1.39	0.05
**phagocytic receptor**				
*Fcgr2b*	Fc fragment of IgG, receptor	Rn00598391_m1	0.61	0.03
*Itgam*	CD11b	Rn00709342_m1	0.57	0.01
**Toll-like receptor**				
*Tlr4*	Toll-like receptor 4	Rn00569848_m1	0.81	0.05
*Tlr9*	Toll-like receptor 9	Rn01640054_m1	0.74	0.07

Relative quantities were the mean of six individual samples. One-way ANOVA was used to evaluate statistical significance. RQ, relative quantity.

### Neuroinflammatory genes regulated by both E2 and isotype selective ER agonists

The isotype selective ER agonists also showed significant transcriptional effects (Table 4). Among the E2-regulated genes nine, including defensins, C3 and its receptor Cd11b, IgG-2a, Ccl2, Il6, RT1-Aw2 and Fcgr2b were regulated similarly by ERα and ERβ agonists (Table 5). In addition, all the three ER agonists evoked up-regulation of mast cell protease Mcpt8 and Mcpt9, and down-regulation of Cd74 and IFN regulatory factor Irf7 (data not shown).

**Table 4:** The effects of ERα agonist 16α-LE2 and ERβ agonist DPN on the transcription of E2-regulated neuroinflammatory genes.

Symbol	Gene name	TaqMan ID	RQ(LE2)	RQ(DPN)
**Complement**				
*C3*	complement C3	Rn00566466_m1	0.743	0.801
**Defensin**				
*Np4*	defensin NP-4 precursor	Rn00597762_g1	2.268	2.578
*RatNP-3b*	defensin ratNP-3 precursor	Rn01478511_gH	10.568	4.574
**Ig chain**				
*IgG-2a*	immonoglobulin G	Rn01429839_g1	9.257	4.048
**Lymphokine**				
*Ccl2*	chemokine (C-C) ligand 2	Rn00580555_m1	0.824	0.703
*Il6*	interleukin-6	Rn00561420_m1	2.232	2.635
**MHC**				
*RT1-Aw2*	RT1 class Ib	Rn03034964_u1	0.658	0.463
**phagocytic receptor**				
*Fcgr2b*	Fc fragment of IgG, receptor	Rn00598391_m1	0.649	0.651
*Itgam*	CD11b	Rn00709342_m1	0.655	0.798

Nine genes were regulated in a similar way by selective ERα and ERβ agonists indicating that both ERs were involved in the transcriptional regulation of these genes. Relative quantities were the mean of six individual samples. RQ, relative quantity; LE2, 16α-LE2; DPN, diarylpropionitrile.

**Table 5:** Summary of estrogenic regulation of neuroinflammatory genes.

Symbol	Gene name	RQ(E2)	RQ(LE2)	RQ(DPN)
**Analogous changes**				
*C3*	complement C3	0.703	0.743	0.801
*Ccl2*	chemokine (C-C) ligand 2	0.527	0.824	0.703
*Fcgr2b*	Fc fragment of IgG, receptor	0.615	0.649	0.651
*IgG-2a*	Ig chain	8.228	9.257	4.048
*Il6*	interleukin-6	2.823	2.232	2.635
*Itgam*	CD11b	0.571	0.655	0.798
*Np4*	defensin NP-4 precursor	2.562	2.268	2.578
*RatNP-3b*	defensin ratNP-3 precursor	13.266	10.568	4.574
*RT1-Aw2*	RT1 class Ib	0.606	0.658	0.463
**E2-specific changes**				
*C4b*	C4	0.670	0.881	0.785
*Cx3cr1*	fractalkine receptor	0.820	0.914	1.041
*Esr1*	estrogen receptor-α	1.392	1.169	0.908
*Mpeg1*	macrophage expressed gene 1	0.611	0.978	0.869
*Tgfb1*	transforming growth factor beta 1	0.779	0.921	0.938
*Tlr4*	Toll-like receptor 4	0.810	0.993	1.069
*Tlr9*	Toll-like receptor 9	0.737	0.896	0.845

The effects of isotype selective ER agonists on the transcription of E2-regulated genes revealed two groups: analogous and specific changes. The large number of analogous changes (genes which are regulated similarly by the three ER agonists) revealed that both ERα and ERβ were involved in the transcriptional regulation of neuroinflammatory genes.

### Genes regulated only by E2

We also found some compound-specific effects. Seven genes, including C4b, Tgfb1, Mpeg1, Cx3cr1, Esr1, Tlr4 and Tlr9 were regulated only by E2 (Table 5).

## Discussion

In this study, we identified the effects of ER agonists on the transcription of neuroinflammatory genes in the frontal cortex of middle-aged female rats. From the major findings we conclude that 1) ERα agonist 16α-LE2 modulates the expression of a large number of genes related to immunity/inflammation, 2) E2, 16α-LE2 and DPN are potent regulators of neuroinflammatory gene expression, 3) estrogens' effects are mediated by both ERα and ERβ, 4) estrogens target glial cells including microglia, 5) estrogens suppress genes encoding key elements of C-mediated phagocytosis, 6) E2 may alter the lymphokine profile, 7) E2 can reverse age-related repression of ERα.

### Expression profiling revealed potent immunomodulatory effects of ERα agonist in the middle-aged female neocortex

In the frontal cortex, ERα agonist 16α-LE2 regulated the expression of numerous genes related to immunity/inflammation. There were similarities between the effects of 16α-LE2 and E2 [20] on immunity/inflammation gene expression. Overlapping effects include down-regulation of C3, Cd74, Fcgr2b and RT1-Aw2. On the other hand, a characteristic feature of the ERα agonist-evoked changes was the up-regulation of genes encoding antimicrobial peptides and S100 proteins. Antimicrobial peptides represent evolutionary ancient weapons of the immune system [36]. Antimicrobial activity of these peptides contributes to the defense mechanism against pathogens. Some of these peptides can chemoattract monocytes and macrophages through CCR2 [37,38]. S100A8 and S100A9 calcium-binding proteins can form a non-covalent heterocomplex which is involved in diverse functions. In macrophages, the complex regulates microtubule reorganization during phagocyte migration [39], NADPH oxidase complex assembly and calcium-dependent signaling during phagocyte activation [40].

Our data indicate that 16α-LE2-induced up-regulation of antimicrobial peptide and S100 protein genes may support defense mechanisms associated with microglia, astrocytes and blood-derived monocytes in the frontal cortex of middle-aged females.

### Estrogens are potent modulators of neuroinflammatory gene expression

Profound regulation of immunity/inflammation genes by 16α-LE2 led us to further investigate the effects of estrogens on additional neuroinflammatory genes of primarily glial origin. We identified sixteen E2-regulated changes including up-regulation of defensin Np4 and RatNP-3b, IgG-2a, Il6 and Esr1, and down-regulation of C3 and C4, lymphokine genes Ccl2 and Tgfb1, MHC gene RT1-Aw2, Mpeg1, Cx3cr1, phagocytic and recognition receptor genes Fcgr2b, Itgam, Tlr4 and Tlr9. These data indicate that decreasing levels of E2 result in a significant change in the expression of neuroinflammatory genes which alters the innate immune response in the frontal cortex of aging females.

The effects of 16α-LE2 and DPN showed similarities to the effects of E2. All ER agonists evoked up-regulation of defensin genes, Il6, and down-regulation of complement C3 and some phagocytic receptors. Up-regulation of defensins and down-regulation of C3 and its receptor Cd11b can modulate various glial cell functions. Up-regulation of Il6 can affect a broad range of processes through the widely expressed IL6R in the cerebral cortex [41].

### Both ERα and ERβ are involved in the immunomodulatory effects of E2

The large number of overlapping genes indicated that both ERα and ERβ were involved in the remarkable immunomodulatory effects of E2. These findings are in accord with published results obtained in *in vitro *[42] and *in vivo *[13,43] LPS and EAE [12] models.

The significant effect of DPN on neuroinflammatory gene expression we found is in agreement with previous results implicating ERβ in the estrogenic regulation of microglia-mediated inflammation [44]. Very recently, an ERβ-specific transrepression pathway has been identified which is controlled endogenously with 5-androsten-3β,17β-diol [45]. This mechanism inhibits inflammatory responses of microglia and astrocytes [45]. These results highlight the potential of selective ERβ agonists to suppress microglia and astrocytes in various neuroinflammatory diseases [46].

### E2 targets glial cells including microglia in the aging frontal cortex

It is known that estrogens influence the regulatory functions of microglia via ERs [13,44,47]. We found several genes, such as Mpeg1 [48], Cx3cr1 [49], Cd11b [50], Tlr4 [51] and Tlr9 [52] which are expressed predominantly in microglia, and were suppressed by E2. Down-regulation of Cd11b is in accord with previous observations showing suppression of microglia reactivity by estrogens [53,54]. It is known that Cd11b expression correlates with microglia reactivity, and accumulating evidence indicates that the microglia phenotype changes during aging [4,55]. In the aged CNS, microglial cells possess elevated reactivity as characterized by up-regulation of cell surface activation markers [55]. Our findings indicate that estrogens suppress microglia reactivity in the aging female cortex. This is consistent with earlier observations that E2 attenuates LPS-induced microglia reactivity in the rat brain [13]. Transcriptional regulation of the fractalkine and toll-like receptors by E2 is novel finding and may have functional consequences. As fractalkine receptor signaling is involved in the regulation of microglia neurotoxicity [56], E2 may alter this microglia function via down-regulation of Cx3cr1.

### E2 may suppress complement-mediated phagocytosis involved in synapse elimination

In the aging female cortex, we demonstrated down-regulation of C3 in the presence of estrogens. This finding is in line with the presence of 3 ERE sequences in the C3 promoter [57,58] and estrogenic regulation of C3 in other tissues [59]. Up-regulation of early C components has been reported recently in the aging mouse forebrain [60]. Following activation, C promotes local inflammation and facilitates destruction through opsonization and lysis [61]. Host tissue is protected from C lysis by soluble and membrane-bound regulators, but cortical neurons express low level of C inhibitors which makes them susceptible to C-mediated damage [62]. As a result of the activation of the classical C pathway, C3b fragment is released from C3, which in turn binds to the surfaces of microbes, apoptotic or injured cells to label them for elimination by professional phagocytes [63]. In the CNS, microglial cells recognize C3b or its proteolytic fragments via multiple receptors including Cd11b/Cd18, which leads to phagocytosis of the labeled substance. This C-mediated mechanism is responsible for the elimination of weak or unwanted synapses in the developing and the aging CNS [63,64]. It is likely that both astrocytes and microglia are involved in this synapse elimination mechanism [65] which is highly relevant to the layer-specific loss of synapses in the estrogen-deprived, aging female neocortex [66].

Our results indicate that the expression of C3 and its receptor Cd11b, and the reactivity of microglial cells are suppressed by estrogens which may contribute to their neuroprotective effects in the cerebral cortex [12,67-69].

### Estrogens may alter the lymphokine profile

We also followed the effects of estrogens on the expression of Ccl2 and Il6. We found down-regulation of Ccl2 by ER agonists which was in agreement with recent data observed in EAE model [70]. On the other hand, we demonstrated up-regulation of Il6 in the frontal cortex of middle-aged rats by E2 and isotype selective ER agonists. This is in line with publications reporting estrogen-responsiveness of Il6 [71]. However, Il6 shows down-regulation by E2 in osteoblastoma Saos-2 cells [72], in contrast to the up-regulation we report in the frontal cortex. Different regulation of Il6 in human osteoblastoma and rodent glial or neuronal cells can be a result of tissue- and species-specificity of estrogen effects [73]. A recent publication reports antimicrobial peptide-induced IL6 expression in glial cells via P2Y receptor signaling [74]. This finding suggests that secondary effects may be involved in the transcriptional regulation of Il6 in a chronic treatment paradigm.

In the CNS, the actions of IL6 are complex and diverse that are mediated by the widely expressed IL6R [41]. IL6 regulates neuroimmune and inflammatory responses [75], neurogenesis [76], neuronal differentiation, growth and survival [77]. As astrocytes are one of the major sources of chemokines and cytokines in the CNS, astrocytes are likely to contribute to the anti-inflammatory effects of estrogens [69,70].

### E2 can reverse age-related repression of ERα transcription

E2 replacement evoked up-regulation of ERα. This finding suggested that chronic treatment with E2 supported estrogen responsiveness of the cortex. It warrants comprehensive examination of the effects of estrogen replacement in various tissues to correctly estimate the benefits and risks of replacement therapies. Up-regulation of ERα has particular importance as ERα expression decreases during aging [78], and it might support the hypothesis of 'critical period' to start an effective hormone replacement in postmenopausal women [79].

## Conclusion

This study provided evidence that E2 and isotype selective ER agonists modulate the expression of neuroinflammatory genes in the middle-aged female frontal cortex. Our results suggest that aging and decreasing level of E2 together result in significant alterations of the innate immune response rendering the middle-aged female brain susceptible for inflammation. The use of ERα agonist therapy in postmenopausal women is hampered by the mammotrophic and uterotrophic activities of ERα agonists. As ERβ agonists have only minor effects in classic estrogen target tissues, we propose that ERβ is a promising drug target to suppress the glial cell response in the E2-deprived female brain and in various neuroinflammatory diseases.

## Abbreviations

C: complement; DPN: diarylpropionitrile; E2: estradiol; ER: estrogen receptor; Ig: immunoglobulin; KEGG: Kyoto Encyclopedia of Genes and Genomes; 16α-lactone-estradiol: 16α-LE2; LPS: lipopolysaccharide; MHC: major histocompatibility complex: PCR: polymerase chain reaction.

## Competing interests

The authors declare that they have no competing interests.

## Authors' contributions

MS, EH, IK, ZL designed the study. MS, EH, IK collected the tissues and isolated RNA. NS, KT, MB were involved in the microarray analysis. MS, EH, IL run and evaluated the real-time PCR. JS, SM synthesized the ERα agonist compound. MS, ZL wrote the manuscript. All authors have read and approved the final version of the manuscript.
